# A large multi-country outbreak of monkeypox across 41 countries in the WHO European Region, 7 March to 23 August 2022

**DOI:** 10.2807/1560-7917.ES.2022.27.36.2200620

**Published:** 2022-09-08

**Authors:** Aisling M Vaughan, Orlando Cenciarelli, Soledad Colombe, Luís Alves de Sousa, Natalie Fischer, Celine M Gossner, Jeff Pires, Giuditta Scardina, Gudrun Aspelund, Margarita Avercenko, Sara Bengtsson, Paula Blomquist, Anna Caraglia, Emilie Chazelle, Orna Cohen, Asuncion Diaz, Christina Dillon, Irina Dontsenko, Katja Kotkavaara, Mario Fafangel, Federica Ferraro, Richard Firth, Jannik Fonager, Christina Frank, Mireia G Carrasco, Kassiani Gkolfinopoulou, Marte Petrikke Grenersen, Bernardo R Guzmán Herrador, Judit Henczkó, Elske Hoornenborg, Derval Igoe, Maja Ilić, Klaus Jansen, Denisa-Georgiana Janță, Tone Bjordal Johansen, Ana Kasradze, Anders Koch, Jan Kyncl, João Vieira Martins, Andrew McAuley, Kassiani Mellou, Zsuzsanna Molnár, Zohar Mor, Joël Mossong, Alina Novacek, Hana Orlikova, Iva Pem Novosel, Maria K Rossi, Malgorzata Sadkowska-Todys, Clare Sawyer, Daniela Schmid, Anca Sîrbu, Klara Sondén, Arnaud Tarantola, Margarida Tavares, Marianna Thordardottir, Veronika Učakar, Catharina Van Ewijk, Juta Varjas, Anne Vergison, Roberto Vivancos, Karolina Zakrzewska, Richard Pebody, Joana M Haussig

**Affiliations:** 1 World Health Organization (WHO) Regional Office for Europe, Copenhagen, Denmark; 2 European Centre for Disease Prevention and Control (ECDC), Solna, Sweden; 3 Global Outbreak Alert and Response Network (GOARN), Geneva, Switzerland; 4 Outbreak Research Team, Institute of Tropical Medicine, Antwerp, Belgium; 5 Centre for Health Security and Communicable Disease Control, The Directorate of Health, Reykjavik, Iceland; 6 Infectious Disease Prevention and Control Unit, Department of Infectious Risks Analysis and Prevention, Centre for Disease Prevention and Control of Latvia, Riga, Latvia; 7 Unit for Diagnostics Preparedness of Notifiable and High Consequence Pathogens, Public Health Agency of Sweden, Solna, Sweden; 8 Field Services, United Kingdom Health Security Agency, London, United Kingdom; 9 Directorate General of Health Prevention, Ministry of Health, Rome, Italy; 10 Santé publique France, the French National Public Health Agency, Saint-Maurice, France; 11 Division of Epidemiology, Public Health Services, Ministry of Health, Jerusalem, Israel; 12 National Centre of Epidemiology, Carlos III Health Institute, CIBER in Infectious Diseases (CIBERINFEC), Madrid, Spain; 13 Health Services Executive, Health Protection Surveillance Centre, Dublin, Ireland; 14 Department of Communicable Diseases, Health Board, Tallinn, Estonia; 15 Infectious Disease Control and Vaccinations Unit, Department of Health Security, Finnish Institute for Health and Welfare, Helsinki, Finland; 16 Communicable Diseases Centre, National Institute of Public Health, Ljubljana, Slovenia; 17 Public Health Wales, Cardiff, United Kingdom; 18 Department of Virus and Microbiological Special Diagnostics, Statens Serum Institut, Copenhagen, Denmark; 19 Department for Infectious Disease Epidemiology, Robert Koch Institute, Berlin, Germany; 20 Ministry of Health, Government of Andorra, Andorra la Vella, Andorra; 21 Surveillance Coordination Department. Hellenic National Public Health Organization (EODY), Athens, Greece; 22 The Norwegian Institute of Public Health, Oslo, Norway; 23 Coordinating Centre for Health Alerts and Emergencies (CCAES), Directorate General of Public Health, Ministry of Health, Madrid, Spain; 24 Department of Microbiological Reference Laboratory, National Public Health Center, Budapest, Hungary; 25 Public Health Service of Amsterdam (GGD Amsterdam), Amsterdam, the Netherlands; 26 Croatian Institute of Public Health, Zagreb, Croatia; 27 National Centre of Surveillance and Control of Communicable Disease, National Institute of Public Health Romania, Bucharest, Romania; 28 Head of Public Health Emergency Preparedness and Response Division, National Center for Disease Control and Public Health, Tbilisi, Georgia; 29 Department of Infectious Disease Epidemiology and Prevention, Statens Serum Institut, Copenhagen, Denmark; 30 Department of Infectious Diseases Epidemiology, National Institute of Public Health, Prague, Czech Republic; 31 Directorate of Information and Analysis, Directorate-General of Health, Lisbon, Portugal; 32 Public Health Scotland, Edinburgh, Scotland, United Kingdom; 33 Directorate of Epidemiological Surveillance and Intervention for Infectious Diseases, Hellenic National Public Health Organization (EODY), Athens, Greece; 34 Department of Communicable Disease Epidemiology and Infection Control, National Public Health Center, Budapest, Hungary; 35 Public Health Services, Ministry of Health, Jerusalem, Israel; 36 School of Health Sciences, Ashkelon Academic College, Ashkelon, Israel; 37 Health Directorate, Luxembourg, Luxembourg; 38 Austrian Agency for Health and Food Safety (AGES), Vienna, Austria; 39 National Institute of Public Health (NIH) - National Research Institute, Warsaw, Poland; 40 Communicable Disease Surveillance Centre, Public Health Wales, Cardiff, United Kingdom; 41 Santé publique France Regional Office, Saint-Denis, Île-de-France, France; 42 Emerging Infectious Diseases Unit, Department of Infectious Diseases, Centro Hospitalar Universitário de São João, Porto, Portugal; 43 Laboratory for Integrative and Translational Research in Population Health (ITR), and EPIUnit - Institute of Public Health, University of Porto, Porto, Portugal; 44 National Program for Sexually Transmitted Infections and HIV Infection, Directorate-General of Health, Lisbon, Portugal; 45 National Institute for Public Health and the Environment (RIVM), Bilthoven, the Netherlands; 46 Department of Communicable Diseases, Health Board, Tallinn, Estonia; 47 ECDC Fellowship Programme, Field Epidemiology path (EPIET), European Centre for Disease Prevention and Control (ECDC), Solna, Sweden

**Keywords:** Monkeypox, MPX, European Region, outbreak, orthopoxvirus

## Abstract

Following the report of a non-travel-associated cluster of monkeypox cases by the United Kingdom in May 2022, 41 countries across the WHO European Region have reported 21,098 cases and two deaths by 23 August 2022. Nowcasting suggests a plateauing in case notifications. Most cases (97%) are MSM, with atypical rash-illness presentation. Spread is mainly through close contact during sexual activities. Few cases are reported among women and children. Targeted interventions of at-risk groups are needed to stop further transmission.

Since detection of monkeypox virus (MPXV) transmission outside endemic areas in May 2022, a large multi-country monkeypox (MPX) outbreak has been ongoing worldwide, with 42,807 cases and 12 deaths reported in 97 Member States across six World Health Organization (WHO) Regions by 23 August 2022 [1]. On 23 July, the WHO Director General declared this outbreak a public health emergency of international concern (PHEIC) [2]. Here we describe the epidemiological features of MPX and analyse disease severity as well as the effect of prior smallpox vaccination on all cases in the WHO European Region reported in TESSy up to 23 August 2022 to inform optimal public health responses.

## Epidemiological situation in the WHO European Region

**Figure 1:**
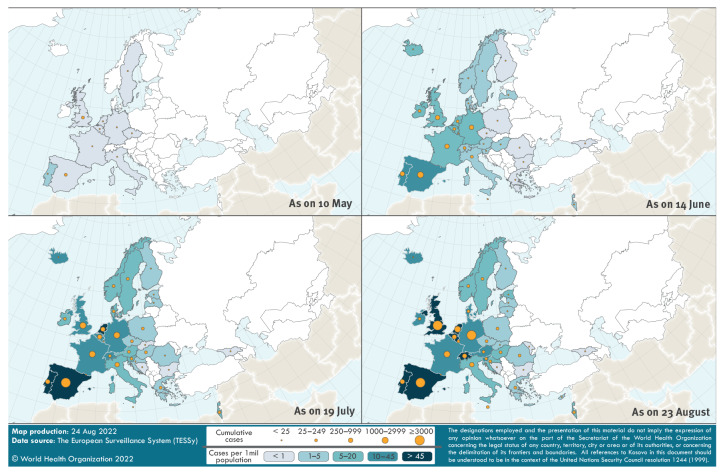
Geographical distribution of monkeypox cases reported through The European Surveillance System (TESSy) by 36 WHO European Region countries, 7 March–23 August 2022 (n = 20,690 cases)

On 13 May 2022, the United Kingdom (UK) reported a non-travel-associated family cluster of MPX cases to the WHO through International Health Regulations (IHR) mechanisms [3]. Thereafter, the UK and other countries, including Portugal, Sweden, Belgium, Germany, Spain, France, Italy, the Netherland, Austria (chronological order) began detecting and reporting MPX cases of Clade II (formerly West African clade) [3,4], primarily among men who have sex with men (MSM). Subsequent retrospective testing of a residual sample in the UK dated the earliest known case back to 7 March 2022. Until end of July [1], Europe remained the epicentre of this large and geographically widespread outbreak, with a steady increase of cases and affected countries (Figure 1).

Distribution of cases by symptom onset or, if missing, the earliest date of diagnosis or notification.

Of 21,098 cases reported in the WHO European Region, case-based data for 20,690 cases (98.1%) from 36 of 41 countries were reported to the European Centre for Disease Prevention and Control (ECDC) and the WHO Regional Office for Europe, through The European Surveillance System (TESSy), using national (n = 9,831 cases) or WHO/ECDC case definitions (n = 1,314 cases) [5,6]. Information is missing or unknown for the other 9,545 cases. Of the total, 99.3% (20,545/20,690) were laboratory-confirmed.

## Nowcasting of monkeypox cases reported in the WHO European Region

**Figure 2:**
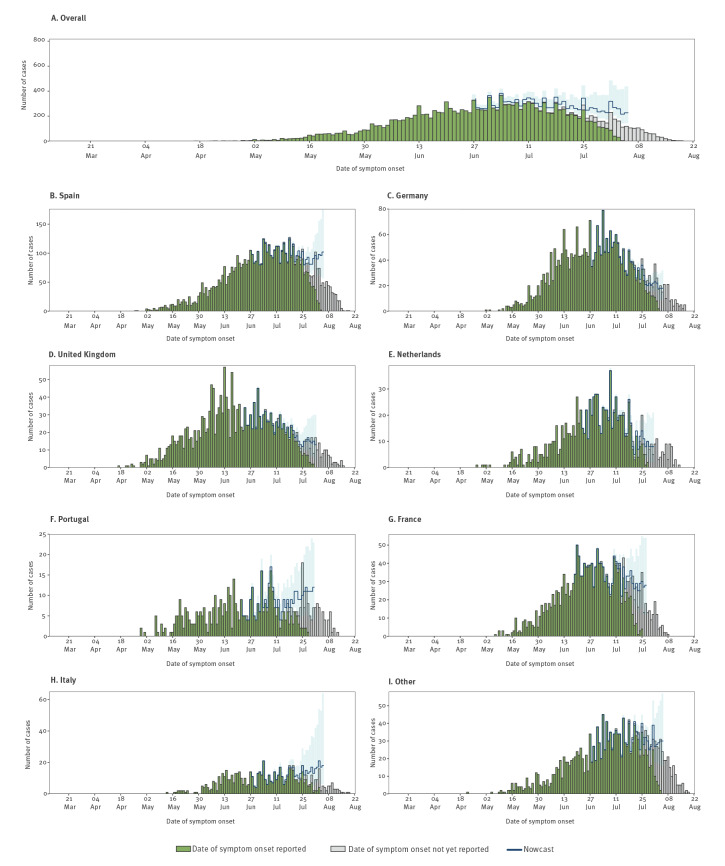
Distribution of reported and nowcasted cases of monkeypox by date of onset of symptoms, 36 WHO European Region countries in order of decreasing incidence, 7 March (week 10)–23 August (week 34) 2022

To assess the current epidemiological situation, we performed nowcasting on TESSy case-based data [7], with a prior negative binomial distribution (mean: 7 days and overdispersion 1.6 days) to adjust for reporting delay, and right truncation at 17 days, which corresponds to 95^th^ percentile of reporting delay for cases in the last weeks. The median reporting delay, defined as the difference in days from date of symptom onset to date of notification at national level, was 7 days (range: 1–117 days) for 17,101 (82.6%) cases with complete date variables. Nowcast estimates suggest that the regional epidemic trend is plateauing overall, with some inter-country differences emerging (Figure 2).

Nowcasting was performed up to 17 days before the last reported date of symptom onset. Reported cases are shown in green. Cases for which the date of symptom onset is not yet in the notification system at the time of nowcasting are shown in grey. Nowcasting point estimate (line) and 95% confidence interval (shaded area) are shown in blue.

Other reporting countries: Andorra, Austria, Belgium, Bosnia and Herzegovina, Bulgaria, Croatia, Cyprus, Czechia, Denmark, Estonia, Finland, Georgia, Greece, Hungary, Iceland, Ireland, Israel, Latvia, Lithuania, Luxembourg, Malta, Norway, Poland, Republic of Moldova, Romania, Slovakia, Slovenia, Sweden, Switzerland.

## Demographic characteristics, clinical presentation and outcome

Most cases (98.8%; 17,685/17,896) identified as male, and the median age of all cases was 37 years (interquartile range (IQR): 31–44; range: 0–88 years) and 37.2% (3,070/8,257) were HIV-positive (Table 1). Among male cases, 96.9% (8,771/9,053) self-identified as MSM. A small proportion of infections have consistently been reported in women and children. In total, 220 adult cases with a known gender were reported to be non-male (1.2%) and 41 cases aged under 18 years (0.2%) have been reported in TESSy. Of these, 15 cases were under 15 years of age.

**Table 1:** Demographic, clinical characteristics and disease-severity of confirmed and probable monkeypox cases, 36 WHO European Region countries, 7 March–23 August 2022, (n = 20,690 cases)

Variables		Overall cases		Hospitalised		Not hospitalised		Unknown		Hospitalisation ratio (per 1,000 cases)	p value
		n	%	n	%	n	%	n	%		
Total cases		20,690	100	197	100	10,601	100	9,892	100	10	
Age group (years)	0–17	41	0.2	2	1.0	25	0.2	14	0.1	49	0.015
	18–30	5,078	24.5	57	28.9	2,504	23.6	2,517	25.4	11	
	31–40	8,231	39.8	87	44.2	4,202	39.6	3,942	39.9	11	
	41–50	4,970	24.0	40	20.3	2,695	25.4	2,235	22.6	8	
	51–60	1,882	9.1	9	4.6	947	8.9	926	9.4	5	
	> 60	442	2.1	2	1.0	209	2.0	231	2.3	5	
	Unknown	46	0.2	0	0.0	19	0.2	27	0.3	0	
Gender^a^	Female	212	1	4	2	137	1.3	71	0.7	19	0.404
	Male	17,685	85.5	193	98	10,457	98.6	7,035	71.1	11	
	Other	16	0.1	0	0	6	0.1	10	0.1	0	
	Unknown	2,777	13.4	0	0	1	0.0	2,776	28.1	0	
Prior smallpox vaccination	Vaccinated	528	2.6	12	6.1	495	4.7	21	0.2	23	0.334
	Not vaccinated	2,974	14.4	94	47.7	2,758	26.0	122	1.2	32	
	Unknown	17,188	83.1	91	46.2	7,348	69.3	9,749	98.6	5	
Smallpox vaccination for current event	PEPV	42	0.2	0	0	40	0.4	2	0	0	0.461
	PPV	1	0	0	0	1	0	0	0	0	
	PEPV/PPV	4	0	0	0	2	0	2	0	0	
	Not vaccinated	3,017	14.6	101	51.3	2,798	26.4	118	1.2	33	
	Unknown	17,626	85.2	96	48.7	7,760	73.2	9,770	98.8	5	
HIV status	Positive	3,070	14.8	37	18.8	2,697	25.4	336	3.4	12	0.441
	Negative	5,187	25.1	52	26.4	4,536	42.8	599	6.1	10	
	Unknown	12,433	60.1	108	54.8	3,368	31.8	8,957	90.5	9	
STI	Yes	93	0.4	8	4.1	81	0.8	4	0	86	0.67
	No	625	3	44	22.3	537	5.1	44	0.4	70	
	Unknown	19,972	96.5	145	73.6	9,983	94.2	9,844	99.5	7	
Sexual orientation	MSM	8,777	42.4	84	42.6	6,677	63	2,016	20.4	10	Not calculated
	Bisexual	93	0.4	4	2	80	0.8	9	0.1	43	
	Heterosexual	276	1.3	9	4.6	242	2.3	25	0.3	33	
	Unknown	11,544	55.8	100	50.7	3,602	34.0	7,842	79.2	13	
Health worker	Yes	64	0.3	0	0	56	0.5	8	0.1	0	0.64
	No	3,645	17.6	80	40.6	3,334	31.4	231	2.3	22	
	Unknown	16,981	82.1	117	59.4	7,211	68	9,653	97.6	7	
Rash	Not reported	657	3.2	4	2.0	424	4.6	229	2.0	6	0.085
	Reported	12,415	60.0	187	94.9	8,367	90.1	3,861	34.4	15	
	Unknown/no data on symptoms	7,618	36.8	6	3.0	494	5.3	7,118	63.5	1	
Lymphadenopathy	Not reported	7,837	37.9	91	46.2	5,118	55.1	2,628	23.4	12	0.005
	Reported	5,235	25.3	100	50.8	3,673	39.6	1,462	13.0	19	
	Unknown/no data on symptoms	7,618	36.8	6	3.0	494	5.3	7,118	63.5	1	
Systemic symptoms^b^	Not reported	4,596	22.2	91	46.2	2,917	31.4	1,588	14.2	20	< 0.001
	Reported	8,476	41.0	100	50.8	5,874	63.3	2,502	22.3	12	
	Unknown/no data on symptoms	7,618	36.8	6	3.0	494	5.3	7,118	63.5	1	

MSM: men who have sex with men; PEPV: Post-exposure preventive vaccination; PPV: Primary preventive (pre-exposure) vaccination; STI: sexually transmitted infection.

^a ^Gender collected in TESSy as female, male, other (e.g. transgender) or unknown.

^b^ Fever, fatigue, muscle pain, chills and/or headache.

Based on case-based data reported in TESSy, hospitalisation ratios and p values were calculated for cases for whom hospitalisation status (i.e. not hospitalised, hospitalised for isolation purposes (n = 129 cases) or hospitalised for clinical management purposes (n = 197 cases)) was known. Cases whose hospitalisation status was reported as unknown or who were known to have been hospitalised, but purpose (isolation/clinical management) was unknown (n = 254) were not included in the analyses. ‘Hospitalisation’ is defined as hospitalisation for clinical care (n = 197 cases). Hospitalisation for known isolation (n = 129 cases) is included as ‘Not hospitalised’. P values were calculated by Fisher’s exact test. For each tabulation of hospitalisation (yes/no) by another variable, when one of the cells was equal to 0, 0.5 was added to all cells of the table in order to be able to conduct the statistical test.

All variables excluding vaccination are up to 23 August 2022. Smallpox vaccination variables combine data from 10 August 2022 and 23 August 2022 for completeness.

Of those reporting symptoms, most reported rash (95.0%; 12,415/13,072) and at least one systemic symptom (64.8%; 8,476/13,072) such as fever, fatigue, muscle pain, chills or headache. Some cases (48.1%; 5,973/12,415 reported rash in the anogenital region; of those, 554 reported no other symptom. Six percent of cases (576/9,732) were hospitalised (n = 129 for isolation purposes; n = 197 for clinical care and n = 250 for unknown reasons). Cases hospitalised for isolation purposes were considered as ‘not hospitalised’ in the analyses. Three cases were admitted to an intensive care unit (ICU) and two of these cases died with encephalitis. 

To estimate predictors of severity, case hospitalisation ratios were calculated. The overall case hospitalisation ratio was 10 per 1,000 cases (Table 1) and did not vary over time (data not shown). Younger cases, those presenting with lymphadenopathy and those without systemic symptoms were at significantly higher risk of hospitalisation (p = 0.015, p = 0.005 and p<0.001, respectively). However, surveillance data does not allow capture of the full clinical course, therefore lack of systemic symptoms at the time of report cannot be interpreted as a predictor of severe disease without further in-depth clinical characterisation. No statistically significant difference was observed for other variables. Firth logistic regressions with hospitalisation as a binary outcome and age as a linear variable showed decreasing odds of hospitalisation with increasing age (odds ratio (OR): 0.97; 95% confidence interval (CI): 0.96–0.99). When considering those hospitalised for unknown reasons, HIV-positive cases were at higher risk of hospitalisation compared with HIV-negative cases (46 and 30/1,000 respectively, p < 0.001) (data not shown). 

## Exposure settings and transmission routes

Detailed data on possible exposure in the 21 days before symptom onset was only available for a minority of cases, limited to some countries. Sexual contact was reported as a possible route of transmission in 93.9% (6,385/6,797) of cases, followed by other person-to-person routes (PTP; non-sexual, non-mother-to-child and non-healthcare associated, 5.3%; 359/6,797) or fomites (0.2%; 11/6,797) (Table 2). Of the cases who reported ‘other’ as a route (0.3%; 41/6,797), 12 also reported likely exposure at a bar event, and one reported household fomite transmission. Many cases reported exposure at a private party/club (69.4%; 2,530/3,643) and/or a large event (28.3%; 1,030/3,643). Household exposure was reported by 233 (6.4%) cases, and these cases also reported sexual transmission (78.1%; 153/196) or PTP (21.4%; 42/196). Likely mode-of-transmission and exposure setting was reported for five cases under 15 years, which indicated transmission through contact with a parent or in the household.

**Table 2:** Exposure settings for monkeypox cases, 36 WHO European Region countries, 7 March–23 August 2022 (n = 20,690 cases)

Variables			Exposure setting^a^ (n = 3,643 cases reporting at least one setting)																						
			Household			Work		School/nursery		Healthcare		Private party/club with sexual activity		Large event with sexual activity		Large event w/o sexual activity		Bar/restaurant w/o sexual activity		Other		Unknown		Missing	
			n	%	n		%	n	%	n	%	n	%	n	%	n	%	n	%	n	%	n	%	n	%
Total cases	20,690	100	233	100	48		100	0	0	0	0	2,530	100	378	100	652	100	199	100	1,007	100	1,008	100	16,129	100
Age group (years)																									
0–17	41	0.2	3	1.3	0		0.0	0	0	0	0	3	0.1	0	0.0	1	0.2	1	0.5	4	0.4	3	0.3	30	0.2
18–30	5,078	24.5	59	25.4	18		37.5	0	0	0	0	633	25.1	82	21.8	152	23.4	43	21.8	243	24.2	262	26.2	3,947	24.5
31–40	8,231	39.8	99	42.7	18		37.5	0	0	0	0	1,033	41.0	184	48.8	290	44.7	82	41.6	440	43.8	438	43.8	6,323	39.3
41–50	4,970	24.0	53	22.8	6		12.5	0	0	0	0	585	23.2	82	21.8	154	23.7	50	25.4	228	22.7	229	22.9	3,907	24.3
51–60	1,882	9.1	14	6.0	5		10.4	0	0	0	0	228	9.0	26	6.9	48	7.4	18	9.1	72	7.2	59	5.9	1,521	9.4
> 60	442	2.1	4	1.7	1		2.1	0	0	0	0	39	1.5	3	0.8	4	0.6	3	1.5	18	1.8	10	1.0	374	2.3
Gender^b^																									
Male	17,685	98.7	214	91.8	48		100.0	0	0	0	0	2,512	99.3	374	98.9	641	98.3	192	96.5	981	97.4	1,000	99.6	13,182	98.7
Female	212	1.2	18	7.7	0		0.0	0	0	0	0	18	0.7	4	1.1	11	1.7	7	3.5	24	2.4	3	0.3	161	1.2
Other	16	0.1	1	0.4	0		0.0	0	0	0	0	0	0.0	0	0.0	0	0.0	0	0.0	2	0.2	1	0.1	13	0.1
Sexual orientation																									
MSM	8,777	75.7	172	86.0	32		86.5	0	0	0	0	2,325	97.7	339	97.7	532	93.5	138	88.5	868	93.0	698	70.6	4,936	67.5
Bisexual	93	0.8	12	6.0	1		2.7	0	0	0	0	21	0.9	4	1.2	13	2.3	5	3.2	20	2.1	16	1.6	35	0.5
Heterosexual	276	2.4	14	7.0	3		8.1	0	0	0	0	30	1.3	4	1.2	23	4.0	13	8.3	35	3.8	44	4.4	147	2.0
Health worker																									
Yes	64	1.7	4	2.4	0		0.0	0	0	0	0	11	1.3	5	1.8	8	1.5	3	1.8	29	3.6	11	1.8	11	0.8
No	3,645	98.3	162	97.6	46		100.0	0	0	0	0	865	98.7	270	98.2	528	98.5	168	98.2	787	96.4	601	98.2	1,285	99.2
Most likely mode of transmission^c^																									
PTP	359	5.3	42	21.4	6		16.2	0	0	0	0	82	5.8	6	2.0	54	10.1	37	98.2	70	8.1	14	2.5	148	3.8
Sexual	6,385	93.9	153	78.1	30		81.1	0	0	0	0	1,341	94.1	292	97.7	475	88.8	131	75.7	791	91.2	547	97.2	3,698	95.3
Fomite	11	0.2	0	0.0	1		2.7	0	0	0	0	0	0.0	0	0.0	0	0.0	0	0.0	3	0.3	2	0.4	6	0.2
Other^d^	41	0.6	1	0.5	0		0.0	0	0	0	0	2	0.1	1	0.3	6	1.1	5	2.9	3	0.3	0	0.0	28	0.7
Sexual and PTP	1	0.0	0	0.0	0		0.0	0	0	0	0	0	0.0	0	0.0	0	0.0	0	0.0	0	0.0	0	0.0	1	0.0

MSM: men who have sex with men; PTP: non-sexual person-to-person transmission; w/o: without.

^a^ Possible exposure in the 21 days before symptom onset. Multiple exposures per case possible.

^b^ Gender collected in TESSy as female, male, other (e.g., transgender) or unknown (not shown in table).

^c^ No cases reported ‘most likely mode of transmission’ as zoonotic, occupational healthcare, occupational laboratory, vertical or transfusion.

^d^ Many cases reporting ‘other’ route of transmission, also reported sexual, PTP or fomite transmission and exposure at a bar etc. (see text). Further details were not provided.

Sixty-four cases were health workers (1.7%; 64/3,708); of these 62 (96.9%) were male and 55 (85.9%) were MSM. While no occupational exposure in the healthcare setting or workplace has been reported through TESSy, three instances of occupational exposure have been reported to the WHO through other routes to date. Other modes of transmission, including zoonotic, vertical and laboratory transmission were not reported for any cases. Possible exposure settings and transmission routes are not mutually exclusive and local outbreak investigations will help identify clear transmission pathways.

## Smallpox vaccination and disease severity

Only 16.8% (3,525/20,960) of cases reported on smallpox vaccination. Of these, most (81.8%; 2,577/3,152) self-reported as both unvaccinated prior to this outbreak and for this outbreak (median age: 36 years; IQR: 30–41), 423 reported receiving a vaccination before this outbreak (median age: 50 years; IQR: 39–56), one reported primary preventive (pre-exposure) vaccination (PPV) (aged 28 years) and 42 reported post-exposure preventative vaccination (PEPV) for this event (median age: 35.5 years; IQR: 30.3–43.8). We assessed the potential effect of prior smallpox vaccination on disease severity and hospitalisation (Table 3). Overall, 197 cases were hospitalised for clinical care, of which 12 cases (11.3%) reported prior vaccination. Firth logistic regressions to assess association between hospitalisation and vaccination were not statistically significant (adjusted OR: 1.07; 95% CI: 0.53–1.97) (Table 3).

**Table 3:** Outcome by prior smallpox vaccination status among monkeypox cases, 36 WHO European Region countries, 7 March–23 August 2022 (n = 3,502 cases)

Variables		Vaccinated		Unvaccinated		Crude OR	95% CI	Adjusted OR	95% CI
		n	%	n	%				
Total cases		528	15.1	2,974	84.9				
Age group (years)	18–30	49	5.7	817	94.3	Ref	Ref	Ref	Ref
	0–17	0	0	10	100	0.79	0.01–6.26	7.95	1.46–30.44
	31–40	94	6.8	1,298	93.2	1.20	0.85–1.73	0.97	0.61–1.57
	41–50	130	16.0	680	84.0	3.17	2.26–4.50	0.87	0.50–1.51
	51–60	189	58.9	132	41.1	23.62	16.56–34.24	0.3	0.08–0.86
	> 60	62	69.7	27	30.4	37.53	22.30–64.83	0.77	0.15–2.61
	Unknown	4	28.6	10	71.4	Not calculated		Not calculated	
Gender^a^	Male	516	15.0	2,927	85.0	Ref	Ref	Not calculated	
	Female	11	19.6	45	80.4	1.43	0.71–2.66		
	Other	1	33.3	2	66.7	3.40	0.31–25.62		
Hospitalisation^b^	Not hospitalised	495	15.2	2,758	84.8	Ref	Ref	Ref	Ref
	Hospitalised	12	11.3	94	88.7	0.74	0.39–1.29	1.07	0.53–1.97
	Unknown	21	14.7	122	85.3	Not calculated		Not calculated	
Health worker	No	253	15.0	1,437	85.0	Ref	Ref	Not calculated	
	Yes	2	4.9	39	95.1	0.36	0.07–1.07		
	Unknown	273	15.4	1,498	84.6	Not calculated			

CI: confidence interval; OR: odds ratio; Ref: reference.

^a^ Gender collected in TESSy as female, male, other (e.g. transgender) or unknown (not shown in table).

^b^ Hospitalisation is defined as hospitalisation for clinical care (n = 197). Hospitalisation for known isolation (n = 129) is included as not hospitalised for clinical care. Regressions were performed for cases for which there was complete data for the specific variables included in each model. Adjusted OR includes hospitalisation as a binary outcome and age (categorical) and vaccination (binary) as explanatory variables. Vaccinated include those vaccinated for smallpox prior to this outbreak.

## Discussion

The MPXV is currently the most prevalent cause of orthopoxvirus infection in humans. MPX outbreaks have previously occurred largely in African countries, where the virus is enzoonotic. However, in recent years, sporadic cases and clusters of MPXV Clade II have occurred in other regions, largely linked to travel from endemic countries or imported animal to human transmission with limited onward human-to-human spread [8-16]. 

Transmission of MPXV is thought to occur primarily through close or direct physical contact with infected lesions, respiratory droplets or contaminated material [17]. Other transmission routes such as zoonotic or mother-to-child have been described [18]. Previously, typical clinical presentation was described as a prodromal phase, with fever, followed by a widespread, centrifugal, evolving maculopustular rash and lymphadenopathy [19]. People living with untreated HIV infection, pregnant women and young children have previously been identified to be at higher risk of severe MPX [20,21]. Epidemiological studies estimated that prior smallpox vaccination provides ca 85% cross-protection against MPXV and reduces the frequency and severity of symptoms [22,23]. However, routine vaccination was discontinued worldwide following the eradication of smallpox in 1980 and effectiveness of vaccination in the current outbreak remains to be assessed.

We describe an on-going multi-country outbreak of MPXV, mainly transmitted among MSM through close physical contact, often during sexual activities. A large proportion of cases (94%) reported sexual transmission, often at gatherings and events which provided the opportunity for amplification through sexual networks. A smaller number of cases were also steadily reported among women and children. Nowcasting estimates suggest that reported cases have plateaued overall in Europe, however, some countries continue to see an increase. Such variation in projections by country may reflect potential differential implementation and impact of local intervention measures.

Clinical presentation in the current epidemic is atypical compared with previous outbreaks [24,25]. Symptoms involve an atypical rash-illness presentation, with a relatively low, but still notable proportion of patients hospitalised. Severe manifestations such as encephalitis have been reported in a small number of cases [26]. This clinical picture may change in the event of spread into populations with increased risk of severe disease, including those with untreated HIV or otherwise immunosuppressed. Further investigations are required to assess disease severity in immunocompromised individuals and other potential vulnerable groups for the current outbreak. We found no evidence that prior smallpox vaccination significantly protects against severe disease and hospitalisation, which raises questions regarding potential waning protection following vaccination over 4 decades ago. As smallpox vaccines are currently rolled out to at-risk individuals, it is essential that studies are undertaken to understand vaccine effectiveness.

This study has some limitations. The analyses are based on surveillance data submitted to TESSy, which are dependent on availability of data at national level and vary in completeness. Indeed, for a number of variables, including vaccination, the level of missing data makes interpretation of analyses challenging. In addition, any clinical data reported in TESSy is of limited scope and will not reflect the full course of disease. Finally, while nowcasting is a valuable tool to account for delays in reporting, interpretation should consider that missing data and misclassification of symptom onset date and varying reporting delays over time can contribute to a considerable uncertainty around these estimates.

## Conclusions

To interrupt transmission of MPXV, identification and testing, management of cases and contacts, targeted risk communication and strong community engagement with affected groups, implementation of targeted public health measures, combined with PPV/PEPV are fundamental [27-30]. However, the transmission patterns of the virus, coupled with the difficulty of tracing multiple often anonymous sexual contacts, likely under-ascertainment of cases, challenges to access and vaccinate priority groups and stigma complicate the public health response. An integrated response with strong collaboration among at-risk groups, communities, public health authorities, and international health organisations is required to overcome these challenges.
